# Neuronal Circuits for Social Decision-Making and Their Clinical Implications

**DOI:** 10.3389/fnins.2021.720294

**Published:** 2021-10-01

**Authors:** Raymundo Báez-Mendoza, Yuriria Vázquez, Emma P. Mastrobattista, Ziv M. Williams

**Affiliations:** ^1^Department of Neurosurgery, Massachusetts General Hospital and Harvard Medical School, Boston, MA, United States; ^2^Laboratory of Neural Systems, The Rockefeller University, New York, NY, United States

**Keywords:** decision making, social cognition, neuroeconomics, psychiatry, neurophysiology, mental health, primates, translational neuroscience

## Abstract

Social living facilitates individual access to rewards, cognitive resources, and objects that would not be otherwise accessible. There are, however, some drawbacks to social living, particularly when competing for scarce resources. Furthermore, variability in our ability to make social decisions can be associated with neuropsychiatric disorders. The neuronal mechanisms underlying social decision-making are beginning to be understood. The momentum to study this phenomenon has been partially carried over by the study of economic decision-making. Yet, because of the similarities between these different types of decision-making, it is unclear what is a social decision. Here, we propose a definition of social decision-making as choices taken in a context where one or more conspecifics are involved in the decision or the consequences of it. Social decisions can be conceptualized as complex economic decisions since they are based on the subjective preferences between different goods. During social decisions, individuals choose based on their internal value estimate of the different alternatives. These are complex decisions given that conspecifics beliefs or actions could modify the subject’s internal valuations at every choice. Here, we first review recent developments in our collective understanding of the neuronal mechanisms and circuits of social decision-making in primates. We then review literature characterizing populations with neuropsychiatric disorders showing deficits in social decision-making and the underlying neuronal circuitries associated with these deficits.

## Introduction

The success of social species is predicated on group living and collective interactions. Living in a group increases individual fitness via several proximal mechanisms, for example, by sharing the costs of foraging and providing information about resources that are otherwise inaccessible to a single individual (Harper, 1982; King et al., 2008). Groups can also defend more successfully from predators than single individuals (Maestripieri, 2008). However, living in a group can also decrease individual fitness during the competition for limited resources. Notwithstanding, the benefits of group living outweigh the costs to an individual’s fitness. **Deciding with whom to interact and how, therefore, is critical for individual survival.**

These social adaptations are tied to our biology, including brain circuitry and function. Mammals’ brains have co-evolved with social living (Dunbar and Shultz, 2007). We can process and integrate both social and environmental information as well as internal physiological cues to make decisions during social interactions. **Currently, there is strong evidence for distinct neuronal circuits that play different roles during social decision-making.** These adaptations, however, can also go awry, as in neuropsychiatric disorders.

Here, we first review recent developments in our collective understanding of the neuronal mechanisms of social decision-making. We then review literature characterizing populations with neuropsychiatric disorders that show deficits in certain aspects of social interactions. Finally, we relate these deficits in social decision-making in neuropsychiatric disorders to the underlying neuronal circuitries.

### Social Decision-Making

Here, we define social decision-making as the process involving decisions that are taken in a context where one or more conspecifics are involved in the decision or the consequences of it. This includes decisions where the outcome is jointly determined by the actors’ actions, either in sequential or simultaneous movements. Decisions where the action of one actor determines the outcome, but the recipient is one or several conspecifics, as in parental behaviors. And situations when the decision-maker chooses between different social stimuli, as in mate choice. Finally, this definition also includes ‘social context’ effects on individual decisions affected by observing others’ choices, as in informational cascades and conformity.

The brain contains adaptive specializations that execute domain-general computations. These computations need to interact with domain-specialized and content-rich expert systems (Cosmides and Tooby, 2013). Here, we consider social decision-making as a domain-specialized system that uses the common (i.e., domain-general) neuronal mechanism of decision making under a social context.

**FIGURE 1:**
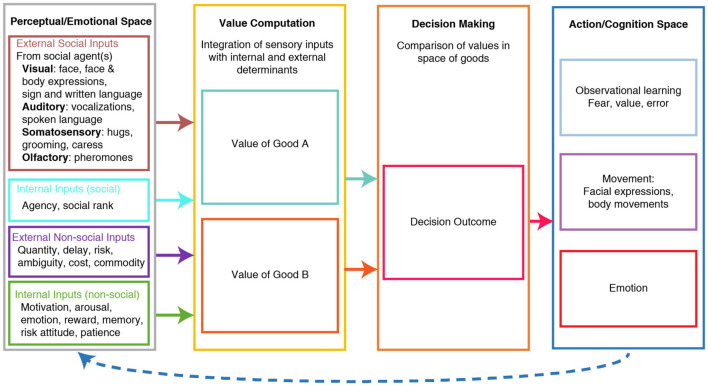
Schema of Value-based social decision-making. Under this model, the value of each good is computed by integrating multiple types of information or determinants, some of which are external social & non-social determinants, while others are social & non-social determinants computed internally. The values of different goods are computed independently of each other and are compared to decide. The decision outcome guides an action plan through a good-to-action transformation. Values and choice outcomes inform future value computations through observational learning, emotion, and observed movements. Based on Padoa-Schioppa (2011).

Social decisions are complex due to several factors. First, an individual’s choice affects not only the agent’s outcome but also others. Second, due to the recursive nature of social interactions, a decision can result in subsequent decisions by others that dynamically modify the subjective values. Third, other’s internal states will affect how they will choose and can only be gleaned through emotional expressions and past behaviors. Fourth, social decisions can be based on another’s knowledge or experience instead of one’s own personal actions/outcomes. Importantly, social decisions can be conceptualized as complex economic decisions. Within this framework, individual social choices are based on subjective preferences between different goods values in a common currency scale. The actor then decides by comparing these goods at the time of the choice, independently of sensorimotor contingencies (Padoa-Schioppa, 2011). This comparison is independent of sensorimotor contingencies because it does not depend on the sensory representation of the good or what action the agent needs to perform to acquire or consume the good. Social factors modify the subjective values of each good and its consumption (Figure 1).

One of the first and fundamental processes in social decision-making is to perceive the other as different agents from the self. This process is so automatic in humans that geometric figures moving in irregular patterns are perceived as alive and volitional agents (Heider and Simmel, 1944; Schultz et al., 2004). To perceive another agent as animated, the agent usually needs to interact contingently and exhibit biological motion. Animacy perception has also been described in non-human primates (Tsutsumi et al., 2012), dogs (Abdai et al., 2017), and birds (Chiandetti et al., 2015).

**During social decisions, other agents’ choices must be considered to make the most advantageous choices for the individual. These considerations, in turn, modify the subjective valuation of different courses of action**. Notably, a decision-maker can optimize the utility derived from its choices using its folk psychology (Dennett, 1983) or theory of mind (Premack and Woodruff, 1978). **The decision-maker considers that other agents are: (1) different from themselves, (2) possess different information about the world, and (3) that this information is used to form their subjective valuations and guide their choices to achieve their goals**. Moreover, the interests of a different agent might go with or against their own.

There is now a large body of experimental evidence supporting the idea that people consider the outcomes of others when making choices (e.g., Fehr and Camerer, 2007). These other-regarding preferences are usually prosocial but can also be anti-social; that is, there can be a positive or negative concern for the welfare of others (Fehr and Schmidt, 1999; Báez-Mendoza et al., 2016). Furthermore, social species also exhibit a strong concern for reputation, affecting an individual’s access to resources (Maestripieri, 2012).

**Social decisions are not restricted to social interactions. During social interactions, there is at least one recursion between agents, while social decisions also take place without recursion.** For example, other’s choices can influence the valuation process via conformity. Social groups can bias the valuation of different courses of action so strongly that they contradict private, or asocial, valuation of the same action (Asch, 1955; Raafat et al., 2009). This effect is most pronounced when a group’s decisions are uninformed (Klucharev et al., 2009). They can even influence individuals to ignore their private information to conform to other group members (Spitzer et al., 2007).

Many scientific disciplines have contributed to our understanding of social decision-making, including psychology, neurobiology, primatology, ethology, ecology, mathematics, and philosophy. Recently, neuroeconomics—the combination of cognitive neuroscience methods with tools used in economics, including game-theoretic tasks based on behavioral economics, has increased our knowledge about social decision-making. Notably, the field of game theory can provide several tools to operationalize social decisions, making them more amenable to study in the laboratory. Game theorists have generated several games that capture different aspects of social decisions. The Dictator Game, Ultimatum Game, Prisoner’s Dilemma, and the Bach-or-Stravinsky game, for example, are now staples of the social neuroscience literature (Rilling et al., 2002). These tasks are associated with theoretical predictions about how different agents should play, which are useful for interpreting the results and accumulating data across studies (Fehr and Camerer, 2007).

Developing a better understanding of the multiple neuronal mechanisms underlying social decision-making will allow us to garner a better understanding of neuropsychiatric disorders. Particularly, for disorders in which social decision-making can be perturbed as autism spectrum disorder, schizophrenia, depression, anxiety, and bipolar disorder.

## Social Decision-Making Neuronal Networks

**Social decisions can be conceptualized as a subset of economic decisions**. The latter involves the comparison of options varying in multiple dimensions based on subjective preferences. Thus, for this type of decision, there is no correct answer. When we choose, values are assigned to the available options, and a decision is achieved by comparing these values and selecting the highest valued good. While the options can vary on multiple dimensions, the value represents a common unit of measure with which to make a comparison (Padoa-Schioppa, 2011). In other words, the brain creates abstract representations of the goods, and the decision-making process involves the computation and the comparison of these values within the space of goods (Padoa-Schioppa, 2011). According to this approach, the valuation of a good is computed when we deliberate and depend on multiple internal and external determinants. This good-based model of decision-making involves the acquisition of sensory information and its integration with external and internal determinants into a subjective value. Decisions are then made comparing these values and the transformation of choice into action. Importantly, the values are not fixed but computed at the time of the choice, and they are independent of the sensorimotor contingencies of choice. Thus, the decision is independent of the offers’ spatial location and of the motor action that will be used to obtain the chosen good. Although this is the theoretical framework we take, this issue is not settled and scientists argue against the concept of a common currency in decision making (e.g., [Bibr B233][Bibr B90]).

The acquisition of social information is shaped by external and internal determinants that influence the computation of its subjective valuation. We hypothesize that social decisions share the comparison mechanisms and decision-to-action transformation of economic decisions (Figure 1).

Social decisions involve the activation of a broad network of cortical and subcortical structures. We think about these circuits as interacting nodes with different specializations; these include the perception of social information, the integration of social information with internal and external processes to estimate subjective values, the comparison of these subjective values, and the transformation of the decision into an action or a cognitive state. In primates, the social decision-making circuit involves the superior temporal sulcus (STS), amygdala, striatum, anterior insula (AI), orbitofrontal cortex (OFC), anterior cingulate (ACC), and prefrontal cortex (PFC). The role of these areas has been characterized using lesion studies, electrophysiology, and fMRI techniques for measuring neural activity during tasks involving social interactions, choices, and valuation.

### Social Information Processing Network

Social information is acquired through different sensory modalities, including audition, touch, olfaction, and vision. For example, human interaction relies on language, while other mammals can learn about potential predators (Cheney and Seyfarth, 1980) or the location of food sources (Hauser, 1992) through vocal communication. The somatosensory system plays a role in providing information about other’s intentions, actions, or pain (Keysers et al., 2010) and is crucial for establishing affective or aversive relations between peers. At the same time, pheromones can elicit various social behaviors (Michael and Keverne, 1968; Isogai et al., 2018). In the following section, we focus on vision as a modality of social information acquisition, its network, and computations (Figure 1, Perceptual space).

#### Face Processing

Living in large social groups obliges primates to decode and value social signals to decide and act. In primates, one of the most informative sources of social signals is the face. Within less than a second, a face conveys information about age, gender, identity, familiarity, mood, gaze direction, and intention (Tsao and Livingstone, 2008). Humans with deficits in facial perception are impaired to recognize faces; thus they experience difficulties during social interactions (Tsao et al., 2006).

Charles Gross and collaborators were the first to discover neurons selective for faces and hands in the temporal cortex of the macaque’s brain (Gross et al., 1972; Bruce et al., 1981; Gross, 2005). Since then, face cells have been found across the temporal (Perrett et al., 1982, 1992) and prefrontal cortices (Rolls et al., 2006) of macaque monkeys. Studies using neuroimaging techniques revealed that face neurons are organized in patches of several millimeters in diameter (Tsao et al., 2003, 2008a; Tsao and Livingstone, 2008). These patches are in the lower bank of the STS and mediodorsally in the fundus. **Monkeys have 6 *face patches* across the temporal lobe, in which single neurons encode information about face orientation, viewing direction, identity, and familiarity** (Tsao and Livingstone, 2008). The adjacent cortex to the *face patches* is modulated by emotional expressions (Hadj-Bouziane et al., 2008), as well as by gaze direction (Morin et al., 2015).

The STS *face patches* form a strong and specific interconnected hierarchical network (Tsao et al., 2008a). They are organized along a posterior-anterior axis, and facial information is transformed from view-specific representations into late, identity abstract representations.

Additionally, there are **three face-selective** patches in the macaque **OFC and dorsolateral prefrontal (dlPFC) cortices** (Tsao et al., 2008b). More recent work has revealed that OFC face neurons —in the lateral sulcus,— encode face dimensions for social categories and emotions like age, gender, and facial expressions (Barat et al., 2018). Moreover, Sliwa and colleagues, using whole-brain fMRI have shown that the posterior lateral OFC, as well as the medial and ventrolateral prefrontal cortices, are active during the observation of social interactions (Sliwa and Freiwald, 2017).

One goal of the face-processing network is to recognize familiar faces. Familiar faces are recognized faster and more accurately than unfamiliar ones when viewing conditions are suboptimal. Familiar faces engage the face-processing network more than unfamiliar ones. Two additional face areas located within the perirhinal cortex and the temporal pole of the macaque monkey are particularly engaged during the observation of familiar faces, suggesting they form part of a familiar face-recognition network (Landi and Freiwald, 2017; Landi et al., 2021).

The human and macaque face processing systems are similar. Like in macaques, humans possess multiple, spatially separated face areas within the temporal and frontal lobes (Duchaine and Yovel, 2015). We have the occipital face area (OFA) of the mid fusiform gyrus (Gauthier and Logothetis, 2000), the fusiform face area (FFA) (Kanwisher et al., 1997), and another area in the posterior part of the temporal sulcus (STS-face area) (Hoffman and Haxby, 2000). Besides, we have a face-selective prefrontal region in the inferior frontal gyrus [IFG-face area, Tsao and Livingstone (2008)]. Similar to the monkey *face patches*, the response to a face is augmented when presented in an anatomically congruent manner with a body (Fisher and Freiwald, 2015).

The amygdala is a collection of subcortical nuclei in the temporal lobe. It contains bilateral face-responsive regions in the dorsal portion of the basal and lateral nuclei. Distinct subdivisions in the amygdaloid nuclei respond differently to the dimensions of a face stimulus. The basolateral (BLA) nucleus is sensitive to valence perception by showing differential responses between a threatening and a neutral facial expression, whereas the central and bed nucleus of the stria terminals responds differently to averted and directed faces (Hoffman et al., 2007). Bilateral amygdala lesions in macaque monkeys eliminate the robust tendency for face viewing preference (Taubert et al., 2018). These results reveal a fundamental role for the amygdala in guiding movements toward face stimuli, a behavior essential for social interaction.

Primate amygdala neurons integrate spatial and motivational information, thus influencing the allocation of resources to relevant stimuli (Peck et al., 2013). Furthermore, unilateral administration of oxytocin to the BLA increased the attention to recipients of reward during prosocial decisions (Chang et al., 2015). This mechanism is crucial for emotional recognition in facial expressions, a behavior that requires direct gaze and attention to relevant parts of the face. SM, a patient with bilateral lesions in the amygdala, showed impairments in recognizing fear facial expressions, as she couldn’t direct her gaze and attention toward emotionally relevant parts of the face, including the eye region (Adolphs et al., 2005). Amygdala lesions cause a heterogenous array of social and non-social deficits -for an extensive review see Gothard (2020). These findings suggest that the **amygdala is not only a perceptual node but *integrates internal and external determinants for driving social decisions and behaviors*** (Figure 1). Crucially, amygdala neurons are multisensory (Morrow et al., 2019) and present multidimensional selectivity properties (Putnam and Gothard, 2019). **Within the amygdala, multiple circuits converge and the same subset of amygdala neurons can be recruited by different neural ensembles combining social and non-social information into high-dimensional representations** (Putnam and Gothard, 2019). Future experiments using, for example, naturalistic social interactions in structured behavioral tasks inspired by game theory will help us address the multiple roles that these nuclei play in social decision making and will expand our understanding of this key brain region.

**FIGURE 2:**
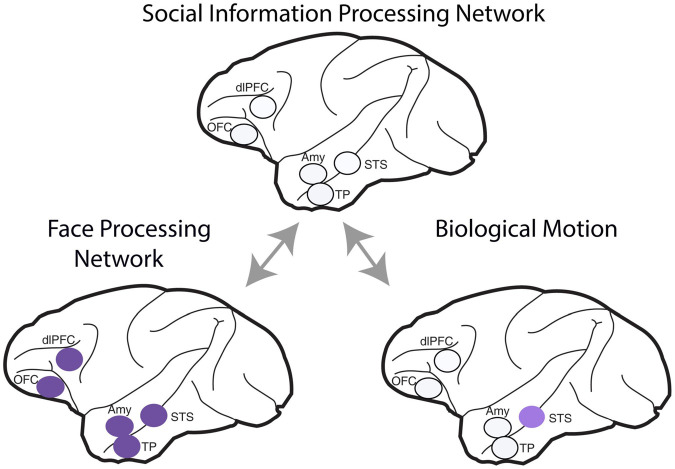
Brain regions that comprise the social information processing network. The face-processing network includes the *face patch* areas along the superior temporal sulcus (STS), the temporal pole (TP), and the amygdala (Amy) within the temporal lobe, the lateral orbitofrontal cortex (OFC) and the dorsolateral prefrontal cortex (dlPFC) in the frontal lobe. Biological motion is encoded by neurons in the superior temporal sulcus (STS). Bidirectional arrows indicate the interdependence of these processes on acquiring social information. The areas are illustrated on a side view of a Rhesus brain.

In macaques, the volume of gray matter within the mid-superior temporal sulcus, inferotemporal cortex, rostral STS, amygdala—all areas involved in perceiving individuals—and rostral PFC correlates with the size of the individual’s troop (Sallet et al., 2011). These findings suggest that the brain has specialized structures dealing with the acquisition and representation of information about conspecifics. In summary, in primates, the **face information processing network** involves several brain regions located in the frontal lobe and along the temporal lobe, including **the amygdala**, **STS, and temporal pole** (Figure 2).

#### Biological Motion

In addition to processing facial information, the STS contains single neurons active during the observation of biological motion, i.e., when perceiving an organism move (Figure 2; Perrett et al., 1985a,b; Perrett et al., 1989, 1992). Neurons within the STS area project to the inferior parietal lobe (IPL) and to the anterior intraparietal cortex (Rizzolatti and Craighero, 2004; Nelissen et al., 2011). Interestingly, these visual neurons in STS do not respond to own movements and do not project to the ventral premotor cortex. Therefore, it has been hypothesized that visual information about observed actions is processed in the STS area, followed by the IPL and finally to the ventral premotor cortex, among other prefrontal areas (Rizzolatti and Craighero, 2004).

The **STS is anatomically connected** (Petrides and Pandya, 2007) to other brain areas implicated in social processing, e.g., the amygdala, OFC, and ACC. This connectivity pattern plays a crucial role in linking facial representations to emotional, motivation, and valuation of social and non-social stimuli.

In the next section, ***we discuss research supporting the role of the amygdala, OFC, ACC, anterior insula, mPFC, and striatum in the integration of internal and external information to compute subjective values for driving social behavior***. We also review research in frontal brain areas describing valuation and choice processes when oneself decision involves another social agent.

### Social Valuation of Goods and Decision-Making Network

The amygdala (Grabenhorst et al., 2012; Chang et al., 2015; Putnam and Gothard, 2019), the OFC (Padoa-Schioppa and Assad, 2006), the anterior cingulate cortex (Kennerley et al., 2011), anterior insula (Mizuhiki et al., 2012; Wittmann et al., 2020), substantia nigra pars compacta (SNc; Lak et al., 2014; Stauffer et al., 2014), and the striatum (Samejima et al., 2005; Lau and Glimcher, 2007) are brain regions where neuronal correlates of valuation of goods have been found during non-social decision-making. ***A fundamental question is whether these areas encode rewards, values, and choice signals related to social stimuli in a similar way as they do for non-social ones. It is highly likely that the mechanisms for non-social and social decision-making are shared at some point in the brain.*** However, **social decisions require the evaluation of benefits and costs not only to oneself but, among other factors, that of others as well.** In the following section, we review research describing the valuation and choice processes in the amygdala, OFC, anterior cingulate cortex, anterior insula, SNc, and striatum under social contexts.

#### Value of Social Information

A crucial first step in any social decision is to identify the social context, recognize the agents and valuate the relevant information to make a choice. In a very clever paradigm, Deaner and colleagues measured how valuable it was for a monkey to watch pictures of conspecifics (Deaner et al., 2005). They pitted a constant amount of juice vs. a variable amount of juice plus the opportunity to observe the picture of a conspecific. The monkeys made their choices depending on the amount of juice offered along with the picture. Thus, if the monkey chose a smaller amount of juice plus the opportunity to watch an image, it strongly indicated that the monkey valued watching the image to at least the difference between offered juice volumes. When the monkey chose with an equal probability between the two alternatives, then the difference in offered juice volume was the subjective value for observing the image, the so-called point of subjective equivalence. Male monkeys valued more looking at dominant monkeys and the hindquarters of female monkeys over looking at subordinate monkeys or a non-salient visual stimulus (Deaner et al., 2005).

OFC neurons are known to code reward value, and they showed distinct coding of reward magnitude or image value, but not both (Watson and Platt, 2012). Thus, suggesting that these neurons do not code reward on a single currency (e.g., in juice volume), but rather as different variables, as had been shown before (Padoa-Schioppa and Assad, 2006; O’Neill and Schultz, 2010).

Lesions of the ACC gyrus (encompassing areas 32, 25, and rostral portion of area 24) diminishes the latency to pick food when another monkey is present in a movie clip compared to monkeys without or with lesions on area 24 or in ventral and OFC (Rudebeck et al., 2006). Based on these findings, the authors suggested that the brain tissue in ACC gyrus is necessary for normal interest in other conspecifics.

Primates living in large social groups have dominant and subordinate individuals (Maestripieri, 2008). Under social contexts, the assessment of the hierarchical social ranks of oneself and others is fundamental for building successful social relationships. Social **hierarchical ranks** are abstract concepts crucial for avoiding fights, mating, and making alliances. Amygdala and OFC represent social rank in macaque monkeys (Azzi et al., 2012; Noonan et al., 2014; Munuera et al., 2018). For example, Munuera et al. showed pictures of familiar monkeys and fractals to monkeys while recording single neuronal activity from their amygdala, OFC, and ACC. A fraction of amygdala neurons showed a linear correlation in activity with the rank of the observed monkeys. Interestingly, the same neuronal population encoding the animals’ rank encoded the reward value of the non-social stimuli (Munuera et al., 2018). This finding is complemented by an MRI study in which the gray matter volume in the amygdala (and raphe nucleus and posterior hypothalamus) was positively correlated with an animal’s social status (Noonan et al., 2014). Thus, the amygdala can represent own and others social status. Furthermore, reciprocal connections between the amygdala and OFC are crucial for OFC neurons to encode value representations (Rudebeck et al., 2013).

Human fMRI studies have consistently identified the amygdala, hippocampus, striatum, ventromedial prefrontal cortex, and the lateral prefrontal cortex in the perception and learning of social dominance (Watanabe and Yamamoto, 2015). For example, activity in the amygdala and rostro medial prefrontal cortex tracked knowledge about a social hierarchy (Kumaran et al., 2012; Ligneul et al., 2016), while learning about one’s position in the hierarchy correlates with activity in the medial prefrontal cortex (Kumaran et al., 2016). Similarly, in the rodent medial prefrontal cortex, the strength of excitatory inputs to pyramidal neurons correlates with the animal’s position in the social hierarchy (Wang et al., 2011), while the activity of these neurons correlates with agonistic effort (Zhou et al., 2017). These results suggest an interplay between the amygdala, striatum, and prefrontal structures in representing a group’s hierarchy and one’s position within in it.

People with OFC lesions struggle to recognize facial expressions (Hornak et al., 2003), make poor social judgments (Damasio et al., 1994; Willis et al., 2010), behave awkwardly in social contexts and face difficulties in value-based decision-making (Fellows and Farah, 2007). Lesion studies in monkeys revealed that OFC damage impairs the assignment and update of stimulus values during value-based decision-making tasks (Walton et al., 2010). Together, these results suggest that the **amygdala and OFC are part of a network involved in the encoding of values under social contexts**.

Approaching others is used to evaluate potential mates, threats, and, in general, acquire social information. The neuronal circuitry involved in this behavior includes the prefrontal cortex, basal ganglia, anterior insula, and hypothalamus (McHenry et al., 2017; Murugan et al., 2017; Rogers-Carter et al., 2018; Engelhard et al., 2019; Pfaff and Barbas, 2019). The medial PFC in rodents shows remarkable responses when approaching others and representing others’ spatial locations (Lee et al., 2016; Murugan et al., 2017).

Not surprisingly, this circuit is regulated by hormones and neurotransmitters (McHenry et al., 2017; Rogers-Carter et al., 2018). Oxytocin-receptor (OTR) heterozygous knockout mice do not habituate to conspecifics (Ferguson et al., 2000). Still, they show wild-type levels of habituation after infusion of oxytocin agonists in the central amygdala (Ferguson et al., 2001). Similarly, blocking oxytocin receptors in the insula disrupts the social approach to juveniles (Rogers-Carter et al., 2018). When humans or monkeys freely viewed conspecific faces, intranasal administration of oxytocin increased the number of fixations to the eye region relative to the mouth region (Dal Monte et al., 2014; Simpson et al., 2014; Chang et al., 2015).

A gap still exists regarding the neural mechanisms by which oxytocin and other neurotransmitters influence social cognition. However, studies in non-human primates (NHP) could bridge the precise circuit-level approach used with rodents and the behavioral, imagining, and clinical studies in humans (Putnam et al., 2018).

Animals choose their mates based on, among other mechanisms, the information they gather from observation. They need to perceive sexual cues that provide information about potential mates, integrate these cues, and discriminate between potential mates (Cummings and Ramsey, 2015). For example, in heterosexual humans, sexual attraction is associated with facial symmetry and shoulder-to-waist or waist-to-hips ratios (Buss and Schmitt, 1993), and observing attractive faces is associated with increased neural activity in the striatum and amygdala (Winston et al., 2007; Chuan-Peng et al., 2020). In mice, the ventromedial hypothalamus, medial preoptic area and bed nucleus of the stria terminalis are involved in mate choice (Chen and Hong, 2018). How these subcortical circuits interact with cortical circuits in acquiring information about potential mates is an open question. Reproduction and sexual behaviors are by definition social behaviors that require the integration of multiple internal and external, social, and non-social determinants and the execution of multiple complex social decisions, for thorough reviews on this topic see Newman (1999); Chen and Hong (2018); Jennings and de Lecea (2020); Prounis and Ophir (2020).

**FIGURE 3:**
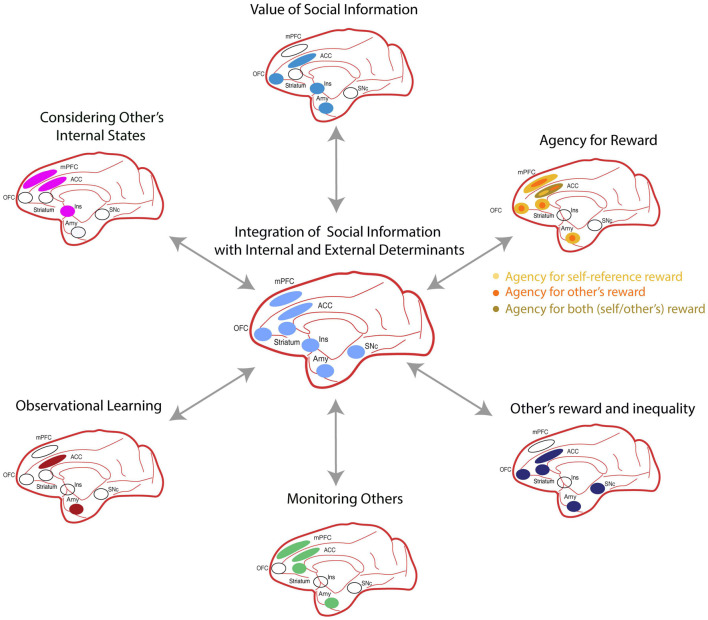
The social valuation network comprises several cortical areas, including: the anterior cingulate cortex (ACC), medial prefrontal cortex (mPFC), medial orbitofrontal cortex (OFC), insula (Ins); and subcortical nuclei including: the anterior striatum, amygdala (Amy), and substantia nigra pars compacta (SNc). Each one of these regions plays a specialized role during distinct cognitive processes of social decision making. Bidirectional arrows indicate the interdependence of these processes on social decisions. All brain regions are illustrated on a sagittal view of a Rhesus brain.

*In summary*, ***acquiring social information is valuable, and the primate temporal, insular, and frontal lobes contain regions specialized in representing this type of social information*** (Figure 3; Tsao et al., 2006; Perrodin et al., 2011). ***Furthermore, this information is then used for valuing different goods.***

#### Agency and Reward

To strategize is to make choices taking into account other players’ strategies (Gibbons, 1992). Most interactions occur many times and knowing what the others did on previous occasions is crucial to strategize. This process is called agency assignment (Wolpert et al., 2003). An agent can be defined as the organism, be it biological or artificial, which initiates an action (Wolpert et al., 2003); in other words, its author. Agency is the mental attribution of an action and its consequences to a particular agent (Georgieff and Jeannerod, 1998; Gallagher, 2000; Farrer and Frith, 2002; Schütz-Bosbach et al., 2006; Tsakiris et al., 2006).

The dmPFC contains neuronal subpopulations projecting to the basal ganglia that differentiate between own action and other’s actions (Yoshida et al., 2011). The striatum is a subcortical brain region crucially involved in motivation and is a general-purpose subcortical area capable of integrating social information into the coding of social action and reward (Báez-Mendoza and Schultz, 2013). Striatal neurons in primates respond to own and conspecific’s movements and to own reward, but not to conspecific’s rewards. Much more interestingly, a large fraction of neurons responded to the conjunction of the social agent, either self or other, and reward (Báez-Mendoza et al., 2013). This neuronal activity may help to assign credit to a social agent when receiving a reward from other individuals.

Human fMRI studies on theory of mind have revealed a network of brain areas activated when differentiating between oneself and others. This network includes the temporoparietal junction (TPJ), precuneus, and mPFC (Saxe and Kanwisher, 2003; Spreng and Grady, 2009; Van Overwalle, 2009; van Veluw and Chance, 2014).

The medial superior temporal sulcus (mSTS) in monkeys has a similar connectivity pattern to human TPJ (Mars et al., 2013) and thus is a candidate homolog area. In a recently published study, Ong and colleagues showed that mSTS neurons hold predictions about others choices in a modified chicken game, they also found an interesting modulation to receiving a reward that hinged on how that reward was obtained (Ong et al., 2021). The mPFC, in particular, may play a role in generating a representation of other’s beliefs. For example, its BOLD activity depends on the number of thought recursions (thinking about what the others might be thinking) a person makes (Xiang et al., 2012). Recently, we showed that single neurons in the human dmPFC hold a representation of other’s beliefs that is clearly distinguishable from their own beliefs (Jamali et al., 2021). Which when combined with a second set of mPFC neurons differentiating between true and false beliefs can give rise to a cellular substrate of Theory of Mind.

#### Other’s Rewards and Inequality

Inequality is a ubiquitous phenomenon. It arises from an asymmetric distribution of resources between two or more conspecifics. The difference in resource distribution can have a negative impact on the utility and subjective value of an object (Fehr and Schmidt, 1999). The disutility from an unequal outcome is different depending on who obtains more resources. When the agent receives more than the conspecific, we speak of advantageous inequality. Conversely, when the agent receives less than the conspecific, we speak of disadvantageous inequality.

Interestingly, humans choose to lower their own payoff so that inequality is smaller, a so-called prosocial behavior. For example, when people donate money to charity, they diminish their wealth so that others can be better off (Harbaugh et al., 2007). Disadvantageous inequality, having less than others, can have a negative effect on behavior. For example, progressive taxation is designed to reduce income inequality by implementing higher taxes on higher earners (Wilkinson and Pickett, 2010).

**FIGURE 4:**
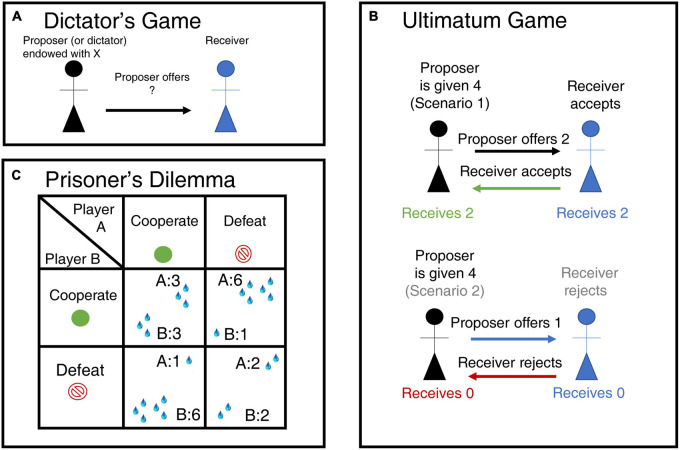
Experimental games used to probe social decision-making. **(A)** Dictator Game. In the Dictator Game the person playing as dictator receives an initial financial endowment and decides to give an amount of the endowment to a receiver. The neoclassical assumption of rational behavior predicts that dictators will not give away anything of their payoff; however, dictators usually give away between 5 and 25% of their initial endowment (Forsythe et al., 1994). It is assumed that the proportion of money given to the receiver is a measure of the disutility for the dictator of having more than the other (Gibbons, 1992; Camerer et al., 2004). **(B)** Ultimatum Game. In this game, the proposer receives an endowment and proposes a split to the responder, just as in the Dictator Game. The responder then either rejects the split, thereby forgoing all monies, or accepts it. Neoclassical economic models predict that the responder will accept any split that results in him having more than nothing. However, responders tend to only accept splits where they obtain more than 30% of the initial endowment (Güth et al., 1982). The responder’s minimum acceptable offer is the percentage of the initial endowment that he is willing to accept 50% of the time (Camerer et al., 2004). This last parameter is directly proportional to the degree of disadvantageous inequality aversion. Some have suggested, instead, that rejecting an offer is a form of altruistic punishment that can foment future cooperation (e.g., Sanfey et al., 2003). **(C)** Prisoner’s Dilemma. In this game, two players choose between cooperation or defection. Mutual cooperation results in a medium-size reward for both players, while mutual defection results in a small reward. But if one of them defects then they receive the highest reward, the tempting reward, while the other receives the lowest, or sucker’s, payoff. The game tests the ability of both players to cooperate and trust others.

The **Dictator Game is commonly used to measure advantageous inequality aversion** (Figure 4 (Forsythe et al., 1994), while the **Ultimatum Game is used to measure disadvantageous inequality aversion** (Figure 4; Güth et al., 1982). Using a **modified version of the dictator’s game** (Figure 4A), in which a monkey can donate or withhold reward to another, Chang et al. (2013) found **three classes of neuronal responses in ACC and OFC that encode the outcomes of social decisions.** Self-referenced neurons responding to **reward outcomes in reference to the self**, i.e., self-received a reward or not. **Other-referenced neurons** signaling the reward only for the other. **Both-referenced neurons signaling the reward for both the self and othe**r in a similar way. Self-reference neurons were found mainly in OFC and anterior cingulate sulcus (ACCs; comprising Broadman area 24c). By contrast, the anterior cingulate gyrus (ACCg; comprising Broadman areas 24a, 24b, and 32) showed a higher proportion of “other referenced” and “both-referenced neurons” than the ACCs or OFC. Orbitofrontal cortex neurons, in particular, had already been shown to respond to other’s rewards (Azzi et al., 2012). Furthermore, the neural activity in the ACCg predicted how prosocial an animal would behave across sessions, but it did not signal the value of the rewards chosen for self and others.

In the same task, BLA neurons signaled the value of the rewards for the self and others when monkeys decided under a social context, but not when there was no conspecific present. Furthermore, the activity of these value-mirror neurons correlates with prosocial decisions (Chang et al., 2015). In a recent study, p**references for rewarding the other monkey increased the synchronization between the BLA and medial prefrontal cortex (mPFC), whereas negative preferences for the other, suppressed the synchronization within the network** (Dal Monte et al., 2020). The results suggest that **social decision preferences rely on mPFC-amygdala network communication**, as well as a different hierarchically layered and computational role between the ACC and the amygdala in this process.

Human fMRI studies have shown that neural activity in the dorsomedial prefrontal cortex (dmPFC) and ventromedial prefrontal cortex (vmPFC) is modulated by **relative subjective value** (Piva et al., 2019). The dmPFC encodes relative subjective value and generalizes across self and others and across different types of tasks involving rewards. This study suggests a role of the **dmPFC** as a node in the frontal lobe that uses **task-invariant mechanisms to compute the relative subjective value for self and others.** Using a similar reward allocation task, Liu and colleagues found that neural activity in the amygdala correlated with the preferred difference in value of a self-other allocation that was augmented with intranasal oxytocin administration (Liu et al., 2019).

Dopaminergic neurons encode subjective value (Lak et al., 2014; Stauffer et al., 2014). Loewenstein and others have long proposed that social motives modify subjective value (Loewenstein et al., 1989; Rabin, 1993; Fehr and Schmidt, 1999). To test if monkeys observing others receive reward affected their subjective valuation of a cue (Noritake et al., 2018) designed a social task for monkey dyads. In this task, a reward was delivered to both members of the dyad following a visual stimulus. These cues predicted reward with different probabilities for each monkey, thus, counterbalancing the expected reward for the self and the other. Interestingly, the subjective value of the self-rewards decreased as the partner-reward probability increased. This result suggests that monkeys show disadvantageous inequity aversion. Critically, this value modulation disappeared when a physical object replaced the conspecific. While the monkeys performed the task, the authors recorded the neural activity in the mPFC and midbrain dopaminergic cells in the ventral tegmental area and substantia nigra pars compacta (VTA/SNc). mPFC neurons monitored the self-reward and other-reward information, whereas the activity of midbrain dopaminergic neurons correlated with the subjective value. The directionality of the information flowed mostly from mPFC to the midbrain. Furthermore, single neurons in the lateral hypothalamus (LH) also reflect the integration of self and other’s rewards and follow the same mPFC to LH information flow (Noritake et al., 2021). Finally, neurons in the striatum hold partial representations of reward inequality between two monkeys (Báez-Mendoza et al., 2016).

Reward inequality results from the comparison of own and other’s rewards when these are of different magnitudes. The results we have reviewed **suggest that the representation of other’s rewards and its comparison to own reward, involve interactions between multiple cortical and subcortical brain structures, including the amygdala, anterior striatum, VTA/SNc, lateral hypothalamus, ACCg, OFC, that seem to be coordinated by the mPFC** (Figure 3).

#### Monitoring Others

During social situations, we need to not only be aware of our own actions but also the actions of the ones we are interacting with. Other’s behavior is salient and can attract our attention and from others’ eye gaze, head, and body orientation, we can infer their focus of attention. Gaze perception, in particular, is the most salient of cues and has been investigated in humans and non-human primates (e.g., Hoffman et al., 2007; Nummenmaa et al., 2010), as it is essential for social cognition and a key to inferring other people’s attention and intentions (Tremblay et al., 2017). Electrophysiological studies in monkeys have identified neurons in the amygdala (Hoffman et al., 2007; Mosher et al., 2014) and the posterior superior temporal sulcus (Shepherd et al., 2006) detecting and tracking the gaze of others. The social attention network is widespread (Freiwald, 2020), and serotonin may play a modulatory role in social gaze behaviors (Weinberg-Wolf and Chang, 2019).

A crucial ability for goal-directed actions and making correct choices is to be able to **detect errors**. Like deriving object-specific reward values or fear learning from social observation, it is known that primates in social settings, can **detect and adjust the value of different choices from their conspecifics**. In humans, the **medial prefrontal cortex (mPFC)** has been implicated in the detection of unfavorable outcomes, response to errors, conflict, and decision uncertainty (Ridderinkhof et al., 2004). In monkeys, single neurons in the mPFC, ventral premotor cortex (PMv or F5), and anterior striatum encode not only their own errors but also those of a different player (Yoshida et al., 2012; Báez-Mendoza and Schultz, 2016; Ninomiya et al., 2020). Furthermore, the action-monitoring neural activity found in mPFC depends on the integrity of signals from PMv (Ninomiya et al., 2020). In the dorsal premotor cortex, a similar cellular representation arises during a turn-taking task, in which monitoring the other’s actions is also paramount for task completion (Cirillo et al., 2018). Thus, the medial prefrontal cortex and its coordination with PMv is part of a cortico-striatal circuit implementing the monitoring of others’ behavior during social interactions.

#### Observational Learning

Primates also learn by observing their social partners. Imagine that you are at your favorite restaurant, and in the process of deciding what to order. You observe a waiter bringing a plate to the neighboring table that you have not tried before. Just watching this menu item increased its subjective value and made it more appealing to order. You then decide to order the dish you just saw. To understand how our brain learns about the rewards of objects by observing our social partners, Grabenhorst and collaborators trained monkey dyads in an observational learning task (Grabenhorst et al., 2019). During this task, the monkeys faced each other over a touch screen. The monkeys took turns to choose between visual objects associated with different reward values shown sequentially. While monkeys performed the task, they recorded single neural activity from the amygdala. They found that while monkeys learn faster from observation, there were three classes of responses in the amygdala. One class of neurons reflected object-specific reward values from observing other’s choices. A second class of neurons coded the difference in value between objects and then converted these values to simulate the partner’s choice. These findings demonstrate that amygdala neurons encode reward values in an abstract way, integrating external information from conspecifics within a social context. Furthermore, these simulation neurons may support an understanding of others’ mental states, as they reflect inferred values and choices from observation.

In primates and rodents, the amygdala and the ACC have been implicated in observational learning and social cognition (Adolphs et al., 2005; Jeon et al., 2010; Klavir et al., 2013). Both brain regions have reciprocal connections (Cassell and Wright, 1986; Carmichael and Price, 1995). In humans, ACCg single neural activity correlates with both the expected outcome and the actual outcome of trials of an observed player (Hill et al., 2016). These responses were different for self-other learning and outcome. While in mice, Jeon and colleagues showed that they developed freezing behaviors—a species-specific response to an aversive stimulus, by observing other mice receiving repetitive foot shocks (Jeon et al., 2010). Furthermore, during observational fear learning, the neural activity in ACC was increased and synchronized with those of the lateral amygdala at a theta rhythm during learning. Recently, (Allsop et al., 2018) and co-workers showed that for observational fear learning to occur, information needs to move from the ACC cortical neurons to the basolateral subdivision of the amygdala during the detection and integration of socially learned cues.

The above **results underscore the role of the ACC in observational learning and highlight the relevance of its functional connectivity with the amygdala in detecting and integrating socially learned information** (Figure 3).

#### Considering Other’s Intentions, Goals, and Actions

The value of possible courses of action is modified when considering other’s intentions, goals, and actions. In a famous example, a flock of ducks distributed itself in two different foraging patches within a pond in order to maximize their individual energy intake (Harper, 1982). The ability to anticipate each other intentions and actions is fundamental for successful social interactions. Several neuroimaging studies using economic **games involving subjects making decisions that affect their payoffs but also the payoffs of other players, find activations in the ACC** (e.g., Tomlin et al., 2006; Xiang et al., 2012).

Single neurons in the monkey ACC encode another individual’s yet unknown decisions during joint interactions during an iterated Prisoner’s Dilemma (iPD; Figure 4C) (Haroush and Williams, 2015) and a modified game of chicken (Ong et al., 2021). Importantly, to optimize the payoff in these tasks it is paramount to monitor the other’s choices. In the iPD, monkeys were on average more likely to engage in mutual cooperation, and a subpopulation of neurons in the **ACC encoded the predicted decision of the other monkey.** Crucially, disrupting cingulate activity with electrical stimulation diminished the rate of mutual cooperation, suggesting a fundamental role of this area in predicting other’s choices and integrating this information in social decisions. Further emphasizing the role of this area in monitoring other’s actions, Hayashi and colleagues recently showed that Japanese macaques predict an agent’s actions independently from their own perspective and, through chemogenetic manipulations, that this ability depends on normal function of the medial frontal cortex (Hayashi et al., 2020).

In the Ultimatum Game, the proposer receives an endowment and proposes a split to the responder (Figure 4). The responder then either rejects the split, thereby forgoing all monies, or accepts it. Neoclassical economic models predict that the responder will accept any split that results in him having more than nothing. However, responders tend only to accept divisions where they obtain more than 30% of the initial endowment (Güth et al., 1982). After an acute tryptophan depletion procedure to temporarily lower 5-HT levels, players were more likely to reject what they perceived as unfair offers (Crockett et al., 2008). Serotonin depletion simultaneously reduced ventral striatal responses to fairness and increased dorsal striatal responses during punishment (Crockett et al., 2013).

Intranasal administration of oxytocin can increase trust behavior in an economic game (Kosfeld et al., 2005), maintain trust behavior following social betrayal, and reduce neural responses associated with the experience of breached trust, including those in the insula (Baumgartner et al., 2008). The anterior insula is involved in considering other’s internal states and expressing emotions. It shows BOLD activity during emotion recognition and expressing emotions (Bartels and Zeki, 2004; Singer et al., 2006), while electrical stimulation of this area in non-human primates can elicit facial expressions of disgust and affiliation (Caruana et al., 2011). The results show that the anterior insula plays a fundamental role in communicative behavior, by integrating multisensory information with internal determinants to induce behavioral responses to external stimuli.

#### Computational Modeling

Computational models of social decision making seek normative accounts of neural and cognitive function. One approach uses reinforcement learning (RL) theory. Under this approach, decisions and learning are described by the unexpectedness of its outcomes. RL models describe how decisions are paired with outcomes over time, by calculating prediction errors for review see Lockwood and Klein-Flügge (2020). RL models can describe decisions both under non-social and social contexts. Looking forward, studies using a reinforcement learning approach could shed light on understanding better the difference in areas tracking social vs non-social prediction errors, as well as the subjective values of choice options (Behrens et al., 2008; Xiang et al., 2013; Pulcu and Haruno, 2020). Crucially, RL models can be applied to behavioral or neural data.

Other models use “recursive sophistication” for understanding mechanisms like theory of mind. This type of model aims to understand how people build internal representations of others during social interactions (Yoshida et al., 2008). More traditional biophysical models aiming to understand the neural and circuit level the mechanisms of SDM in the brain have provided useful insight into how value neurons, and self-other discriminating social neurons can interact, and provide inputs into two different social decision making systems related to self-choice and the simulation of a partner’s choice (Deco et al., 2013; Grabenhorst et al., 2019).

There is now a strong development in the computational modeling of the behavioral and neuronal processes underlying social decisions. This effort can be encapsulated by computational psychiatry. The field of computational psychiatry attempts to systematically conceptualize psychiatric disorders in computational terms (Montague et al., 2012). One of the earliest uses of this approach was the computational phenotyping of people with the autism spectrum disorder while playing the trust game (e.g., Xiang et al., 2012). In summary, further advancement and refinement on the available computational models of social decision making are needed to shed light on the strategies available to interacting individuals as well as their neuronal mechanisms supporting them.

### Summary

In summary, the valuation of goods under social contexts recruits a network that involves several brain areas, including the amygdala, striatum, anterior insula, ACC, and OFC. These areas compute rewards under social contexts and encode abstract categories related to social stimuli, like social hierarchy, gender, identity, and age. Critically, this network overlaps in computing value for non-social stimuli. Notwithstanding, current evidence suggests that there are neuronal subpopulations specialized in encoding social values and categories. Put together, we found evidence that social decision-making is a domain-specialized system that uses the domain-general neuronal mechanism of decision making under a social context (Cosmides and Tooby, 2013).

One critical step in social decision making is the integration of social determinants of value with non-social determinants to compute the value of different goods (Figure 1). Homeostatic changes are internal determinants of value that impact attention, motivation, decision-making, and behavior (Gao and Horvath, 2007; Morton et al., 2014; Tononi and Cirelli, 2014). Emergent evidence suggests the anterior insula, amygdala, mPFC, and OFC play an important role in computing value by integrating external and internal, social and non-social inputs (Padoa-Schioppa, 2011; Gogolla, 2017). The insular cortex in particular possesses extensive anatomical connections that allow integration of multisensory and bodily state information (Droutman et al., 2015), making it a strong candidate region within a neural network for the integration of social and non-social determinants of value.

The regions of the brain valuation system are connected to the decision-making network in a way that information about value and choice are shared in a dynamical and context-dependent manner. The decision-making nodes conformed of OFC, ACC, PFC, and mPFC, encode fundamental decision-making variables under social contexts. Including the relative subjective value of the reward to oneself and to another social agent, as well as the agency to the recipient of the reward when the decisions involve another. At the same time, these areas are involved in observational learning from other social agent’s fear (BLA-ACC), value (amygdala-mPFC), and errors (ACC-mPFC). Future research is required for a better understanding of how interactions and information flow within and across these areas influence social decision-making.

### Future Directions

There is a critical need to understand the neuronal mechanisms of social decision making. Social behavior pervades almost all aspects of our lives. Our ability to interact with others affects interpersonal (Kennedy and Adolphs, 2012), economic (Dixit, 2014), and group dynamics (Wokler, 2001). Similarly, deficits in social behavior are a prominent feature of many neurocognitive disorders, see “Clinical Implications” section. Notwithstanding its relevance to public health, we lack detailed knowledge of the neuronal mechanisms underscoring social decision making. Experiments involving multi-scale recordings in animals and intracortical neurophysiology in humans during social decision-making tasks are powerful tools that will allow us to interrogate how interactions and information flow among brain areas determine social decision making.

Moving forward, the field of social decision making should profit from the emergence of dynamical and contingent social stimuli containing higher levels of naturalism, e.g., avatars, virtual reality settings, as well as movies involving conspecifics and social interactions, to design novel paradigms simulating contexts where social interactions, learning and decision making occur under more natural settings [for review see Fan et al. (2021)]. Moreover, the field should push for behavioral paradigms involving dyads, triads and groups of conspecifics interacting socially and taking decisions in a dynamic and contingent way. In this vein, economic games are ideal paradigms that allow social decision making to be studied experimentally by simulating real-life social interactions in a controlled environment. These games are effective tools for assessing fundamental social traits like valuation, cooperation, trust, altruism, and the influence of others on our decisions. Crucially, humans and NHP can engage in a wide range of economic games allowing us to assess the neural correlates and circuits involved in simple and complex social traits. Experiments involving simultaneous recordings of behavior and neural activity during social and non-social decision making are needed to better understand the nature of the mechanisms and circuits involved in social decision making and how they relate to other decisions.

The above combined with multi-scale and wireless recordings in NHP, as well as non-invasive techniques in humans, and novel tools for measuring natural behaviors and data analysis in semi-natural environments—i.e., through neuroecology—will reveal insights into the neural correlates of social learning, decision making and emotions under ethologically relevant conditions.

There are several key open questions about value computation (Figure 1). For example, where and how are social and non-social determinants of value computed and integrated? How do social and non-social determinants influence each other? And how are the determinants weighted? Future experimental designs need to decompose how specific brain regions appear to contribute to different cognitive processes simultaneously. One such approach is the adaptation of game-theoretic paradigms for neurophysiological study. Not only there are computational models established for specific tasks, but the decisions taken in these tasks are dependent on other’s choices. For example, the iterated prisoner’s dilemma (Haroush and Williams, 2015) incorporates two crucial properties: the outcome is contingent upon the mutual concurrent decisions of both individuals and both decisions can be either concordant or discordant. Therefore, the key to succeeding in the game relies on one’s ability to anticipate the other’s decisions. More importantly, this dissociation of self and other decisions, concordant and discordant interactions, and the dissociation between one’s decision and reward, allows the explicit dissociation of neuronal signals that encode self and other decisions, past responses, social context and expected reward.

While comparison across species should be done carefully (Rushworth et al., 2013), transgenic mice models along with optogenetic tools provide valuable information about the mechanisms and circuits involved in social choices and behavior. Furthermore, computational, and behavioral models of social choices are needed to shed light on the strategies available to interacting individuals as well as their neuronal mechanisms supporting them (Krakauer et al., 2017; Grabenhorst et al., 2019). Moreover, research in the genetics of neurodevelopmental disorders offers a window into the genes, circuits, and mechanisms of social decision-making, as well as potential therapies for mental disorders impairing social decision-making (Sahin and Sur, 2015). Finally, the emerging field of transgenic manipulation in NHP (Liu et al., 2016; Stauffer et al., 2016; Tremblay et al., 2020) opens an avenue of research for monitoring the subpopulation of neurons within micro-circuits, as well as the possibility of better understanding how deficits in the social decision-making network are associated to neuropsychiatric disorders.

## Clinical Implications

The impairment of social decision-making in distinct psychiatric disorders emphasizes the extent to which a distributed neuronal circuitry underlies social decision-making. While disorders such as Depression, Autism Spectrum Disorder, Schizophrenia, Bipolar Disorder, and Social Anxiety Disorder manifest their unique symptomology, they also share different levels of disrupted social decision making. Furthermore, there are efforts underway to develop measurable biomarkers based on social decision making tasks to assess distinct constructs of the research domain criteria as they relate to these disorders (Robson et al., 2020).

### Major Depressive Disorder

Major Depressive Disorder (MDD) affects approximately 16.1 million Americans and is the leading cause of disability in the United States among those aged 15–44. Those suffering from MDD most often experience anhedonia, apathy, fatigue, altered sleep and appetite, and disrupted cognition (McNamara and Houston, 1986; American Psychiatric Association, 2013). The etiology of MDD appears to be partially genetic, while other non-genetic factors can play a role such as viral infections, stress, trauma, or abnormal brain development (Nestler et al., 2002; Sandi and Haller, 2015).

As much as MDD can result in negative feelings surrounding self-perception, it can also have a devastating impact on how individuals form, maintain, and generate interest in social interactions. The common symptoms include a significantly reduced drive for social affiliation, decreased pleasure from social interactions, increased sensitivity to social rejection, and even loss of emotional reactivity to positive social stimuli (Kupferberg et al., 2016).

The Ultimatum Game is often used to investigate behavioral and neuronal responses to fairness and social decision-making (Figure 4B; Güth et al., 1982). Multiple studies have examined the differences in how individuals with depression make decisions during this game (Harle et al., 2010; Destoop et al., 2012; Radke et al., 2013; Scheele et al., 2013).

Patients diagnosed with MDD playing the Ultimatum Game are more likely to reject unfair offers compared to controls (Radke et al., 2013; Scheele et al., 2013; Wang et al., 2014). In a study combining facial expressions with the Ultimatum Game, MDD patients and controls participated in the game as responders. During the game, the offers were accompanied by the emotional facial expressions of the proposer. When proposers’ faces conveyed unambiguous signals of unfair offers, both depressed and control individuals shared higher offer rejection rates. Notably, however, depressed patients rejected more offers than healthy volunteers overall (Radke et al., 2013).

Earlier research suggested that depressed individuals showed increased negative emotional reactions to unfair offers yet accepted them more often than control subjects in the Ultimatum Game (Figure 4B; Harle et al., 2010). By contrast, when they play as proposers, their rate of rejection as responders did not differ from healthy controls (Destoop et al., 2012). The results suggest that changing the framing of the game affects the subjects’ responses. Finally, patients with depression made significantly lower charitable donations compared to controls regardless of the personal cost in one variation of the Dictator Game (Figure 4A) which also reported higher guilt after receiving unfair offers from proposers in the Ultimatum Game (Pulcu et al., 2015).

Several studies have identified brain areas involved in the social and behavioral deficits associated with depression. In the presence of negative emotional stimuli, the amygdala, hippocampus, insula, brainstem regions, and dorsal and ventral prefrontal cortex are more active (Williams, 2016). However, in depressed individuals, this circuit is less active than in healthy controls (Sheline et al., 2010; Stuhrmann et al., 2011; Arnone et al., 2012).

While these responses indicate a dysregulation in the processing of social information and reward response, additional studies conducted using game theory reveals more about the deficit in social decision-making that depressed individuals experience. Increasing the fairness of the offers is associated with increased activation in the striatum in normo-typical individuals. By contrast, patients with depression fail to show this activation (Gradin et al., 2015).

**FIGURE 5:**
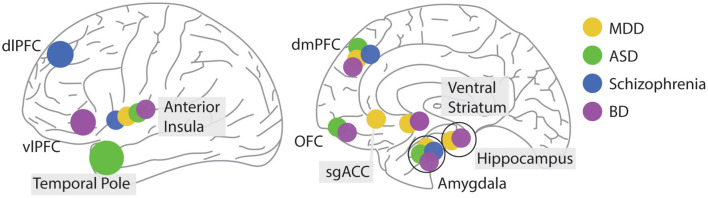
Location of differential brain activity during social decision making associated with different psychiatric conditions. Brain regions are illustrated on a lateral (left) or sagittal (right) view of the human brain.

Even after the remission of MDD, patients show disrupted functional connectivity during social decision-making tasks. In fully remitted, unmedicated patients previously diagnosed with MDD, Pulcu and colleagues studied neural responses during a charitable donation using fMRI. During prosocial decisions, the septal/sgACC region was more active than controls (Pulcu et al., 2014). Abnormal amygdala responses in MDD implicate its role in regulating emotional face perception during social decision-making (Pulcu and Elliott, 2015). Facial emotion recognition in MDD patients is impaired for basic emotions except sadness (Dalili et al., 2015), a process underscored by the amygdala nuclei (Adolphs et al., 1994, 1998; Morris et al., 1996). The amygdala is hyperactive upon presentation of sad stimuli and social information that elicits shame, which likely affects subsequent social decision-making (Tangney et al., 1992; Roy et al., 2009; Stuhrmann et al., 2011; Pulcu and Elliott, 2015; Pulcu et al., 2015; Figure 5).

Overall, patients with depression show disrupted motivation to engage in social interactions due to decreased subjective value derived from them. This change in processing also affects how depressed patients engage in social decisions. They are more likely to reject unfair offers and are less likely to engage in prosocial behaviors. This behavioral process is reflected in decreased reward responses in the ventral striatum, a structure long associated with reward processing during social interactions (Báez-Mendoza and Schultz, 2013). Furthermore, abnormal amygdala functioning can affect a person’s ability to compute the value in social situations (Grabenhorst et al., 2019).

### Autism Spectrum Disorder

Autism Spectrum Disorder (ASD), typically diagnosed during early childhood, consists of core behavioral disturbances including abnormal social behavior, difficulties in verbal and non-verbal communication, and hyper-specific interests associated with repetitive behaviors (Geschwind and Levitt, 2007). Perception of animacy is disturbed in some children with ASD (Congiu et al., 2010). On top of communicative challenges, individuals with ASD face additional difficulties such as having a lack of understanding of social cues and conventions, disinterest in forming new relationships, and difficulties in conversation such as interpreting jokes, sarcasm, or understanding metaphors (Weston, 2019). These impairments tend to result in anxiety, social withdrawal, dysphoric emotions, and high-stress levels.

One of the first processes to be engaged during social decision-making is the acquisition of information about the participants in an interaction, and this process is disrupted “in those diagnosed with ASD”. Subgroups of patients diagnosed with ASD show deficits in face recognition, similar to those shown by prosopagnosics (Barton et al., 2004). This deficit might trickle down to problems in complex decisions. One study investigated the effect of social and non-social visual cues incorporated into an Ultimatum Game (Ikuse et al., 2018). In neutral, non-social conditions healthy controls playing as proposers offered less money to recipients compared to social cues. Participants diagnosed with ASD, however, did not show the same decrease in offer quantity, despite distributing more money overall compared to controls; thus, displaying a potential decreased susceptibility to social cues.

When acting as the recipient in the Ultimatum Game, individuals in the spectrum are more likely to accept unfair offers compared to neurotypicals (Phillips et al., 2003; Tei et al., 2018). Children diagnosed with ASD playing the Ultimatum Game exhibit significantly lower rejection levels for both fair and unfair offers (Hartley and Fisher, 2018). These children were also observed to accept more unfair offers than controls. As for explaining such behavior, accepting unfair offers still results in a physical reward compared to rejection despite the lack of inequality aversion. Similarly, decreased reciprocity rates may correlate with a lack of desire to follow or defend social norms (Hartley and Fisher, 2018; Weston, 2019). The behavioral strategies of adults diagnosed with ASD playing a dynamic Stag Hunt Game, a game requiring coordination with the other player, were better characterized by computational models with lower degrees of belief inference than those of healthy controls (Yoshida et al., 2010). The latter results confirm a deficit in Theory of Mind during social interactions in this clinical population.

There are four main brain areas known to play a role in the symptomology of ASD: the amygdala, OFC, insula, and the temporo-polar cortex (Weston, 2019). Notably, reduced inter-connectivity between the amygdala and insula shown in ASD has been linked to social abnormalities from their involvement in the salience network, which integrates cognitive and social information (Figure 5; von dem Hagen et al., 2013).

### Schizophrenia

Schizophrenia is a debilitating disorder that if uncontrolled, can dismantle all aspects of an individual’s social life (Patel et al., 2014). While the disorder can present a broad range of manifestations, most typically symptoms are described as being positive, negative, or cognitive (Patel et al., 2014). Delusions, hallucinations, anhedonia, and impaired communicative abilities are some of the symptoms that can interfere with an individual’s ability to interact with others (Lehman et al., 2004; American Psychiatric Association, 2013; Patel et al., 2014).

During social decisions, agents need to consider that others possess different information than themselves about the world that guides their decisions, a precursor ability of Theory of Mind. People with schizophrenia show deficits in Theory of Mind tasks (Shamay-Tsoory et al., 2007; Sprong et al., 2007; Billeke et al., 2015; Kronbichler et al., 2017), that is associated with a reduced BOLD response in dmPFC when considering other’s beliefs (Kronbichler et al., 2017).

Judging trustworthiness is essential in repeated interactions for guiding the formation of beneficial relationships and avoiding harmful ones, and this process begins by looking at other faces. In a task that required participants to determine the trustworthiness of presented faces, schizophrenics showed reduced mediolateral OFC and amygdala activation, and significant variability in trustworthiness judgments compared to controls (Baas et al., 2008). In particular, the amygdala and insula have been shown to be significantly associated with facial trustworthiness (van’t Wout and Sanfey, 2008; Santos et al., 2016). Thus, suggesting disrupted processing of trustworthiness while acquiring social information.

Schizophrenic patients playing the Ultimatum Game (Figure 4B) as proposers are consistently more likely to make hyper fair offers, i.e., offers larger than 50% of the initial endowment, than healthy controls (Billeke et al., 2015; Horat et al., 2018). Importantly, hyper fair offers are suboptimal relative to the game’s equilibrium and reveal a lack of trust in the responder.

While playing as receivers in the Ultimatum Game, schizophrenics reject unfair offers at the same rate as healthy controls (de la Asuncion et al., 2015; Horat et al., 2018), but also see Csukly et al., 2011. However, if the proposers are depicted with angry faces on a computer screen, healthy controls tend to reject unfair offers more often, while schizophrenic patients are unaffected (de la Asuncion et al., 2015). Recent studies highlight individual differences among the population diagnosed with schizophrenia. Patil and colleagues report a lower acceptance rate of unfair offers in people with schizophrenia, but similar acceptance rates to healthy volunteers for fair and hyper fair offers, while being unaffected by the emotion expressed by the proposer (Patil et al., 2020).

The data so far suggests that the process of estimating and updating trust in others is disrupted in schizophrenia. This disruption can affect their ability to engage in social decisions, particularly when estimating future actions by their counterparts. Consequently, this disrupted processing affects how they value different options. The activity in the amygdala, in particular, appears to be disrupted in this condition during social decision-making (Figure 5).

### Bipolar Disorder

The classic picture of bipolar disorder (BD) is like a modified sine wave, with mood fluctuating between episodes of mood elevation (mania) and depression, interspersed with periods of euthymia (Harrison et al., 2018). Bipolar disorder encroaches upon psychosocial functioning by altering emotional and social cognition as reflected by increased risky behaviors, sociability, and aggression (American Psychiatric Association, 2013; Grande et al., 2016); yet, its relationship with social decision making, in particular, has not been investigated thoroughly.

Body language is a unique and often subliminal form of communication that can convey feelings and allow an observer to generate predictions based on their movements. Individuals diagnosed with BD had significant impairment in perceiving emotion from body movements compared to healthy controls (Vaskinn et al., 2017). Interestingly, individuals at high risk of developing BD, compared to those with low risk expressed higher rates of approach behavior toward strangers in the Judgment and Context Task (Campellone et al., 2018). Finally, patients with BD playing the Ultimatum Game as a recipient are more likely to reject moderately unfair offers compared to controls (Duek et al., 2014; Lois et al., 2020). These results suggest that people at risk of and with BD show a number of differences in social decision making compared to healthy controls: increased value of social rewards, impairment in perceiving emotion, and heightened sensitivity to fairness.

There are two parallel neuronal circuits affected in BP. A dysfunctional prefrontal-hippocampal-amygdala bilateral circuit is active during emotion processing and regulation. A second circuit encompassing the ventral striatum together with the OFC and the ventrolateral prefrontal cortex is “overactive” during reward-processing in the left hemisphere (Phillips and Swartz, 2014). Interestingly, BD is associated with abnormalities in white matter tracts of the genu of the corpus callosum (Wise et al., 2016). In one of the few studies examining neuronal responses using fMRI during social decision making in euthymic patients diagnosed with BD, Lois and colleagues observed hypoactivation in the right anterior insula in response to unfair offers and a relative hypoactivation compared to the healthy controls in the left ventrolateral prefrontal cortex after rejecting an offer (Lois et al., 2020). Besides the better studies role of dysregulated voltage-gated calcium channels in the phenomenology of bipolar disorder (Harrison et al., 2018); together, these results begin to suggest a role of altered neuronal processing in two brain circuits involved in reward processing and social decision making in this disorder (Figure 5).

### Social Anxiety Disorder

People with social anxiety disorder (SAD), or social phobia, fear and avoid the scrutiny of others (Stein and Stein, 2008). This disorder can be conceptualized in our framework as the consequence of a disrupted negative valuation of social interactions. Testing this hypothesis is part of our collective future work. To date, there are few investigations on how people diagnosed with social anxiety disorder make decisions in structured game-theoretic tasks. In an intriguing study, researchers observed lower levels of oxytocin in plasma of people diagnosed with SAD after playing a single-shot trust game compared to healthy controls (Hoge et al., 2012). It is unknown how this population played the one-shot trust game, however, individuals with SAD playing the trust game iteratively through a computer showed similar behavior as healthy controls (Sripada et al., 2013). In this task, healthy controls exhibited an increased BOLD contrast in the ventral striatum between a partner’s cooperation and a partner’s defection that discriminated between the type of partner. This differential response was absent in the SAD group (Sripada et al., 2013). Although it is not clear if these responses were related to disrupted computation of reward prediction errors during “social” interactions, these results suggest altered neuronal processing of social feedback in the ventral striatum.

### Summary

Performance in social decision making tasks, and particularly in game-theory-based games, differ between healthy controls and psychiatric populations, and between psychiatric diagnoses in a number of ways. See review by Robson and colleagues for a deeper review of this topic (Robson et al., 2020). Two areas show consistent results across diagnoses: (i) impaired ToM and integration of social and cognitive processes, which result in less effective and flexible decision making. (ii) Increased risk avoidance of negative social interactions and reduced reward sensitivity, which results in reduced profit-seeking. While two areas show different effects across diagnoses: (i) emotional reaction to negative interactions is more negative in depression and bipolar disorder, especially compared to anxiety. (ii) Mixed evidence for cooperative and pro-social behavior.

## Conclusions

The success of primate species is predicated on the interactions between conspecifics and our ability to engage in social decision-making. Social decisions particularly affect the valuation of the different courses of action and their outcome. The brain’s valuation neuronal circuitry operates on any good, social, or non-social in nature. But it can be affected by our capacity to acquire social information, learn by observing other actions and errors, and differentiate between our own actions and thoughts and those of others. Reward inequality occupies and drives much of our actions in the social realm (Rabin, 1993; Piketty and Saez, 2014). While considering other’s intentions modifies the value of the goods we pursue. These processes can also go awry, as in neuropsychiatric disorders. Game-theoretic tasks based on behavioral economics are extremely useful to shed light on the neural computations and behaviors involved during social decision-making and how they can be affected in different neuropsychiatric disorders. Developing a better understanding of the multiple neuronal mechanisms underlying social decision-making will allow us to understand neuropsychiatric disorders better.

## Author Contributions

RB-M, YV, EPM, and ZMW wrote the manuscript. All authors contributed to the article and approved the submitted version.

## Conflict of Interest

The authors declare that the research was conducted in the absence of any commercial or financial relationships that could be construed as a potential conflict of interest.

## Publisher’s Note

All claims expressed in this article are solely those of the authors and do not necessarily represent those of their affiliated organizations, or those of the publisher, the editors and the reviewers. Any product that may be evaluated in this article, or claim that may be made by its manufacturer, is not guaranteed or endorsed by the publisher.
